# 4-Amino-5-(2-hydroxy­benzyl­idene­amino)benzene-1,2-dicarbonitrile

**DOI:** 10.1107/S160053680901157X

**Published:** 2009-04-08

**Authors:** Yan Cheng, Jing Gao

**Affiliations:** a Department of Pharmacy, Mudanjiang Medical University, Mudanjiang, 157011, People’s Republic of China

## Abstract

A new tetra­dentate unsymmetrical Schiff base, C_15_H_10_N_4_O, has been synthesized from 4,5-dicyano-*o*-phenyl­enediamine and *o*-vanillin in refluxing ethanol. The dihedral angle between the two benzene rings is 39.0 (1)°. There are intra­molecular O—H⋯N and weak inter­molecular N—H⋯O and N—H⋯N inter­actions.

## Related literature

For the biological activity of Schiff bases, see: Boskovic *et al.* (2003 ▶); Koizumi *et al.* (2005 ▶); Oshiob *et al.* (2005 ▶). For related structures, see: Kannappan *et al.* (2005 ▶); Zhang *et al.* (2003 ▶).
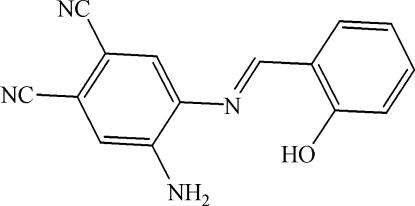


## Experimental

### 

#### Crystal data

C_15_H_10_N_4_O
*M* *_r_* = 262.27
Monoclinic, 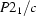

*a* = 14.0158 (15) Å
*b* = 12.3650 (13) Å
*c* = 7.3557 (8) Å
β = 99.904 (2)°
*V* = 1255.8 (2) Å^3^
*Z* = 4
Mo *K*α radiation
μ = 0.09 mm^−1^
*T* = 273 K
0.12 × 0.10 × 0.08 mm

#### Data collection

Bruker APEXII CCD area-detector diffractometer
Absorption correction: multi-scan (*SADABS*; Bruker, 2001 ▶) *T*_min_ = 0.989, *T*_max_ = 0.993
7234 measured reflections
2838 independent reflections
1770 reflections with *I* > 2σ(*I*)
*R*_int_ = 0.027

#### Refinement

*R*[*F* ^2^ > 2σ(*F* ^2^)] = 0.046
*wR*(*F* ^2^) = 0.170
*S* = 1.00
2838 reflections
187 parameters
2 restraints
H atoms treated by a mixture of independent and constrained refinement
Δρ_max_ = 0.14 e Å^−3^
Δρ_min_ = −0.16 e Å^−3^

### 

Data collection: *APEX2* (Bruker, 2004 ▶); cell refinement: *SAINT-Plus* (Bruker, 2001 ▶); data reduction: *SAINT-Plus*; program(s) used to solve structure: *SHELXS97* (Sheldrick, 2008 ▶); program(s) used to refine structure: *SHELXL97* (Sheldrick, 2008 ▶); molecular graphics: *SHELXTL* (Sheldrick, 2008 ▶); software used to prepare material for publication: *SHELXTL*.

## Supplementary Material

Crystal structure: contains datablocks I, global. DOI: 10.1107/S160053680901157X/fl2237sup1.cif

Structure factors: contains datablocks I. DOI: 10.1107/S160053680901157X/fl2237Isup2.hkl

Additional supplementary materials:  crystallographic information; 3D view; checkCIF report

## Figures and Tables

**Table 1:** Hydrogen-bond geometry (Å, °)

*D*—H⋯*A*	*D*—H	H⋯*A*	*D*⋯*A*	*D*—H⋯*A*
N3—H1*B*⋯N2^i^	0.930 (18)	2.205 (17)	3.126 (3)	171 (2)
N3—H1*C*⋯O1^ii^	0.930 (19)	2.69 (2)	3.206 (3)	115.5 (17)
O1—H1*A*⋯N4	0.82	1.91	2.639 (2)	147

Symmetry codes: (i) 
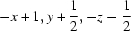
; (ii) 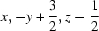.

**Fig. 1.**
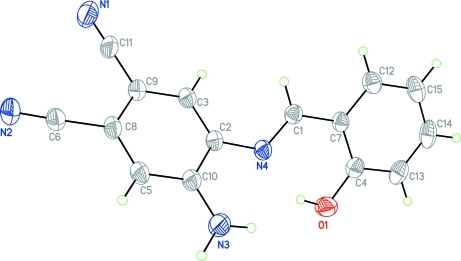
A view of the structure of (I), showing the atmoic numbering scheme and 30% probability displacement ellipsoids.

## supplementary crystallographic information

### Comment

During the past decades, Schiff bases have been intensively investigated not only because of their strong coordination capability but also due to their diverse biological activities, such as antibacterial, antitumor, *etc*. (Koizumi *et al.*, 2005; Boskovic *et al.*, 2003; Oshiob *et al.*, 2005). The halide groups in schiff base ligands could effectively optimize the properties of the coordination complexes.

X-ray diffraction analysis indicates that (I) is an unsymmetrical Schiff base ligand (fig. 1). The imide bond length 1.283 (2)(2) Å for C(1)–N(4) is slightly longer than that of 4-Bromo-2-(2-pyridylmethyliminomethyl)phenol (1.269 (4) Å) (Zhang *et al.*, 2003).  It is noteworthy that there exists relatively weak intermolecular interactions involving the NH moieties and one intramolecular interaction with OH as the donor (Table 1), which are similar to those of its derivative 4-Bromo-2-(2-pyridylmethyliminomethyl)phenol (Zhang *et al.*, 2003).

### Experimental

(I) was prepared according to the method reported in the literature (Kannappan *et al.*, 2005). 4,5-dicyano-*o*-phenylenediamine (2.16 g, 0.02 mol) was added to a stirred ethanol solution of O-vanillin (3.04 g, 0.02 mol (10 ml). The reaction mixture was stirred about 3 h and then the mixture was allowed to stand at room temperature for about two days. Yellow cystals suitable for X-ray diffraction analysis were then collected with a yield of 60%.

### Refinement

The H atoms of the amino group were located from difference density maps and were refined with distance restraints of d(N–H) = 0.93 (2) Å. H atoms bound to C and O atoms were visible in difference maps and were placed using the HFIX commands in *SHELXL97*. All H atoms were allowed for as riding atoms (C–H 0.97 Å, O–H 0.86 Å) with the constraint *U*_iso_(H) = 1.5*U*_eq_(methyl carrier), 1.5*U*_eq_(O) and 1.2*U*_eq_(carrier) for all other H atoms.

### Crystal data

**Table:**

C_15_H_10_N_4_O	*F*(000) = 544
*M**_r_* = 262.27	*D*_x_ = 1.387 Mg m^−^^3^
Monoclinic, *P*2_1_/*c*	Mo *K*α radiation, λ = 0.71073 Å
Hall symbol: -P 2ybc	Cell parameters from 1728 reflections
*a* = 14.0158 (15) Å	θ = 2.2–26.3°
*b* = 12.3650 (13) Å	µ = 0.09 mm^−^^1^
*c* = 7.3557 (8) Å	*T* = 273 K
β = 99.904 (2)°	Block, yellow
*V* = 1255.8 (2) Å^3^	0.12 × 0.10 × 0.08 mm
*Z* = 4	

### Data collection

**Table:**

Bruker APEXII CCD area-detector diffractometer	2838 independent reflections
Radiation source: fine-focus sealed tube	1770 reflections with *I* > 2σ(*I*)
graphite	*R*_int_ = 0.027
φ and ω scans	θ_max_ = 27.5°, θ_min_ = 1.5°
Absorption correction: multi-scan (*SADABS*; Bruker, 2001)	*h* = −18→15
*T*_min_ = 0.989, *T*_max_ = 0.993	*k* = −16→11
7234 measured reflections	*l* = −9→9

### Refinement

**Table:**

Refinement on *F*^2^	Primary atom site location: structure-invariant direct methods
Least-squares matrix: full	Secondary atom site location: difference Fourier map
*R*[*F*^2^ > 2σ(*F*^2^)] = 0.046	Hydrogen site location: inferred from neighbouring sites
*wR*(*F*^2^) = 0.170	H atoms treated by a mixture of independent and constrained refinement
*S* = 1.00	*w* = 1/[σ^2^(*F*_o_^2^) + (0.105*P*)^2^] where *P* = (*F*_o_^2^ + 2*F*_c_^2^)/3
2838 reflections	(Δ/σ)_max_ = 0.001
187 parameters	Δρ_max_ = 0.14 e Å^−^^3^
2 restraints	Δρ_min_ = −0.16 e Å^−^^3^

### Special details

**Table:**

Geometry. All e.s.d.'s (except the e.s.d. in the dihedral angle between two l.s. planes) are estimated using the full covariance matrix. The cell e.s.d.'s are taken into account individually in the estimation of e.s.d.'s in distances, angles and torsion angles; correlations between e.s.d.'s in cell parameters are only used when they are defined by crystal symmetry. An approximate (isotropic) treatment of cell e.s.d.'s is used for estimating e.s.d.'s involving l.s. planes.
Refinement. Refinement of *F*^2^ against ALL reflections. The weighted *R*-factor *wR* and goodness of fit *S* are based on *F*^2^, conventional *R*-factors *R* are based on *F*, with *F* set to zero for negative *F*^2^. The threshold expression of *F*^2^ > σ(*F*^2^) is used only for calculating *R*-factors(gt) *etc*. and is not relevant to the choice of reflections for refinement. *R*-factors based on *F*^2^ are statistically about twice as large as those based on *F*, and *R*- factors based on ALL data will be even larger.

### Fractional atomic coordinates and isotropic or equivalent isotropic displacement parameters (Å^2^)

**Table:**

	*x*	*y*	*z*	*U*_iso_*/*U*_eq_	
C1	0.91817 (12)	0.53191 (15)	0.1796 (2)	0.0451 (5)	
H1	0.9143	0.4649	0.1213	0.054*	
C2	0.75504 (12)	0.55664 (15)	0.0486 (2)	0.0447 (5)	
C3	0.72462 (12)	0.45035 (16)	0.0422 (3)	0.0467 (5)	
H3	0.7640	0.3982	0.1084	0.056*	
C4	1.01931 (14)	0.66149 (16)	0.3888 (3)	0.0495 (5)	
C5	0.60770 (13)	0.60523 (16)	−0.1573 (3)	0.0513 (5)	
H5	0.5687	0.6568	−0.2258	0.062*	
C6	0.48595 (14)	0.46643 (17)	−0.2707 (3)	0.0517 (5)	
C7	1.00930 (12)	0.56357 (15)	0.2900 (2)	0.0433 (5)	
C8	0.57855 (12)	0.49920 (16)	−0.1632 (3)	0.0478 (5)	
C9	0.63646 (13)	0.41989 (15)	−0.0612 (3)	0.0487 (5)	
C10	0.69528 (13)	0.63652 (15)	−0.0496 (3)	0.0491 (5)	
C11	0.60376 (14)	0.30994 (18)	−0.0585 (3)	0.0585 (6)	
C12	1.09021 (14)	0.49709 (18)	0.2966 (3)	0.0551 (5)	
H12	1.0840	0.4315	0.2338	0.066*	
C13	1.10904 (16)	0.69001 (19)	0.4891 (3)	0.0629 (6)	
H13	1.1160	0.7542	0.5559	0.075*	
C14	1.18721 (16)	0.6233 (2)	0.4895 (3)	0.0685 (7)	
H14	1.2472	0.6437	0.5554	0.082*	
C15	1.17898 (14)	0.5268 (2)	0.3944 (3)	0.0671 (7)	
H15	1.2327	0.4824	0.3962	0.080*	
N1	0.57748 (15)	0.22349 (18)	−0.0536 (3)	0.0888 (7)	
N2	0.41430 (13)	0.43718 (16)	−0.3475 (3)	0.0680 (6)	
N3	0.72491 (14)	0.74135 (15)	−0.0435 (3)	0.0700 (6)	
N4	0.84233 (10)	0.59191 (12)	0.1583 (2)	0.0462 (4)	
O1	0.94357 (11)	0.72904 (11)	0.3901 (2)	0.0692 (5)	
H1A	0.8950	0.7034	0.3268	0.104*	
H1B	0.6802 (13)	0.7949 (13)	−0.087 (3)	0.080*	
H1C	0.7813 (10)	0.7570 (18)	0.039 (3)	0.080*	

### Atomic displacement parameters (Å^2^)

**Table:**

	*U*^11^	*U*^22^	*U*^33^	*U*^12^	*U*^13^	*U*^23^
C1	0.0428 (10)	0.0454 (10)	0.0451 (10)	−0.0030 (8)	0.0018 (8)	0.0023 (8)
C2	0.0359 (9)	0.0493 (11)	0.0464 (10)	−0.0008 (8)	0.0002 (8)	−0.0008 (8)
C3	0.0373 (10)	0.0505 (11)	0.0491 (11)	0.0030 (8)	−0.0015 (8)	0.0021 (8)
C4	0.0475 (11)	0.0520 (11)	0.0461 (11)	−0.0053 (9)	0.0000 (8)	0.0062 (9)
C5	0.0389 (10)	0.0566 (12)	0.0540 (12)	0.0045 (9)	−0.0047 (8)	0.0050 (9)
C6	0.0411 (11)	0.0574 (12)	0.0544 (12)	0.0012 (9)	0.0019 (9)	−0.0047 (9)
C7	0.0367 (10)	0.0496 (11)	0.0412 (10)	−0.0016 (8)	−0.0003 (7)	0.0078 (8)
C8	0.0355 (10)	0.0589 (12)	0.0464 (11)	0.0001 (8)	−0.0006 (8)	−0.0024 (8)
C9	0.0391 (10)	0.0527 (12)	0.0522 (11)	−0.0018 (8)	0.0019 (8)	−0.0026 (8)
C10	0.0408 (10)	0.0495 (11)	0.0539 (11)	0.0007 (8)	−0.0004 (8)	0.0031 (9)
C11	0.0426 (11)	0.0556 (13)	0.0708 (15)	−0.0043 (10)	−0.0088 (10)	−0.0018 (10)
C12	0.0479 (11)	0.0643 (13)	0.0514 (12)	0.0038 (9)	0.0036 (9)	0.0068 (9)
C13	0.0586 (13)	0.0691 (14)	0.0556 (13)	−0.0205 (11)	−0.0052 (10)	0.0022 (10)
C14	0.0441 (12)	0.0998 (19)	0.0557 (13)	−0.0192 (12)	−0.0083 (10)	0.0163 (13)
C15	0.0405 (11)	0.0969 (19)	0.0615 (14)	0.0076 (11)	0.0022 (10)	0.0150 (12)
N1	0.0665 (13)	0.0643 (14)	0.124 (2)	−0.0132 (11)	−0.0155 (12)	0.0018 (12)
N2	0.0467 (10)	0.0788 (14)	0.0724 (13)	−0.0042 (9)	−0.0071 (9)	−0.0107 (10)
N3	0.0556 (11)	0.0517 (11)	0.0917 (15)	−0.0012 (9)	−0.0185 (10)	0.0126 (10)
N4	0.0375 (8)	0.0481 (9)	0.0490 (9)	0.0002 (7)	−0.0036 (7)	0.0033 (7)
O1	0.0627 (10)	0.0562 (9)	0.0816 (11)	0.0064 (7)	−0.0076 (8)	−0.0126 (8)

### Geometric parameters (Å, °)

**Table:**

C1—N4	1.283 (2)	C7—C12	1.395 (3)
C1—C7	1.445 (2)	C8—C9	1.405 (3)
C1—H1	0.9300	C9—C11	1.436 (3)
C2—C3	1.380 (3)	C10—N3	1.360 (3)
C2—C10	1.411 (3)	C11—N1	1.133 (3)
C2—N4	1.414 (2)	C12—C15	1.376 (3)
C3—C9	1.387 (2)	C12—H12	0.9300
C3—H3	0.9300	C13—C14	1.371 (3)
C4—O1	1.352 (2)	C13—H13	0.9300
C4—C13	1.390 (3)	C14—C15	1.378 (3)
C4—C7	1.407 (3)	C14—H14	0.9300
C5—C8	1.372 (3)	C15—H15	0.9300
C5—C10	1.396 (3)	N3—H1B	0.930 (18)
C5—H5	0.9300	N3—H1C	0.930 (19)
C6—N2	1.124 (2)	O1—H1A	0.8200
C6—C8	1.456 (2)		
			
N4—C1—C7	123.06 (17)	C3—C9—C11	120.39 (17)
N4—C1—H1	118.5	C8—C9—C11	120.79 (16)
C7—C1—H1	118.5	N3—C10—C5	121.11 (18)
C3—C2—C10	119.77 (16)	N3—C10—C2	119.97 (17)
C3—C2—N4	123.04 (16)	C5—C10—C2	118.88 (17)
C10—C2—N4	117.10 (16)	N1—C11—C9	178.9 (3)
C2—C3—C9	121.21 (17)	C15—C12—C7	121.2 (2)
C2—C3—H3	119.4	C15—C12—H12	119.4
C9—C3—H3	119.4	C7—C12—H12	119.4
O1—C4—C13	118.65 (19)	C14—C13—C4	119.9 (2)
O1—C4—C7	121.79 (16)	C14—C13—H13	120.1
C13—C4—C7	119.57 (19)	C4—C13—H13	120.1
C8—C5—C10	120.72 (18)	C13—C14—C15	121.5 (2)
C8—C5—H5	119.6	C13—C14—H14	119.2
C10—C5—H5	119.6	C15—C14—H14	119.2
N2—C6—C8	176.6 (2)	C12—C15—C14	119.1 (2)
C12—C7—C4	118.76 (17)	C12—C15—H15	120.5
C12—C7—C1	119.65 (18)	C14—C15—H15	120.5
C4—C7—C1	121.57 (17)	C10—N3—H1B	118.9 (14)
C5—C8—C9	120.58 (16)	C10—N3—H1C	116.1 (14)
C5—C8—C6	121.05 (17)	H1B—N3—H1C	122 (2)
C9—C8—C6	118.34 (17)	C1—N4—C2	120.57 (16)
C3—C9—C8	118.78 (17)	C4—O1—H1A	109.5

### Hydrogen-bond geometry (Å, °)

**Table:**

*D*—H···*A*	*D*—H	H···*A*	*D*···*A*	*D*—H···*A*
N3—H1B···N2^i^	0.93 (2)	2.21 (2)	3.126 (3)	171 (2)
N3—H1C···O1^ii^	0.93 (2)	2.69 (2)	3.206 (3)	116 (2)
O1—H1A···N4	0.82	1.91	2.639 (2)	147

Symmetry codes: (i) −*x*+1, *y*+1/2, −*z*−1/2; (ii) *x*, −*y*+3/2, *z*−1/2.
